# Time-domain terahertz emission spectroscopy on van der Waals materials

**DOI:** 10.1557/s43579-026-00940-z

**Published:** 2026-04-22

**Authors:** Claudia Gollner, Aaron Lindenberg, Tony F. Heinz

**Affiliations:** 1 Stanford Institute for Materials and Energy Sciences, SLAC National Accelerator Laboratory, Menlo Park, CA 94025 USA; 2 Department of Applied Physics, Stanford University, Stanford, CA 94305 USA; 3 Department of Materials Science and Engineering, Stanford University, Stanford, CA 94305 USA

**Keywords:** 2D materials, Heterostructure, Interface, Layered, Nonlinear effects, Optical properties, Optoelectronic, Thin film, Transportation, van der Waals

## Abstract

**Abstract:**

Time-domain terahertz (THz) emission spectroscopy provides a direct method to probe transient photo-currents by recording the emitted terahertz electric field. Although the basic principles of THz surface emission have been understood for more than 30 years, the constant progress in ultrafast laser science to ever shorter pulses, the development of new materials and enhanced sensitivity promote THz emission spectroscopy as a reliable method to gain insights into charge carrier dynamics with unprecedented precision. It provides a versatile tool to study ultrafast processes, such as plasmon-driven hot carriers, dynamics of Dirac fermions, interfacial charge transfer, coherent phonon emission and quantum beating, to name only a few. However, despite the rapidly growing body of research on van der Waals materials, especially in their low-dimensional limit, THz emission spectroscopy has only been applied to a limited extent in these material systems. In this prospective, we review time-domain THz emission spectroscopy as a complementary approach to probe ultrafast charge carrier dynamics and the material’s nonlinear response. After a description of the experimental method, we report on THz emission spectroscopy of bulk and 2D van der Waals materials with special focus on graphene and transition metal dichalcogenide layers.

**Graphical abstract:**

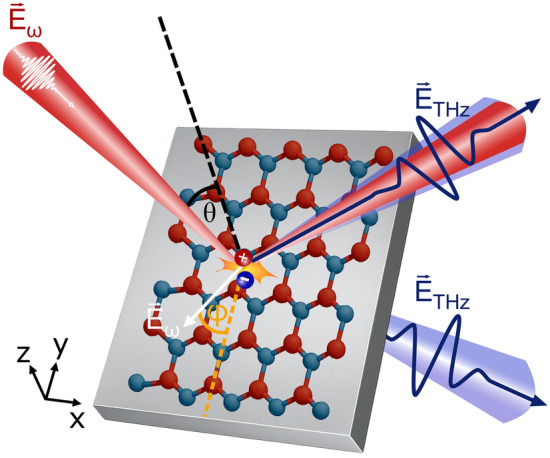

## Introduction

Since the first isolation of graphene by exfoliation in 2004,^[1]^ the field of atomically thin 2D materials has grown into a vast research area. Initial research highlighted the remarkable properties of semi-metallic graphene, including its high carrier mobility, mechanical flexibility, and high optical transparency. For other purposes, the presence of a band gap is important, which was realized in various 2D semiconductors. The discovery of monolayer group-VI transition metal dichalcogenide (TMD) semiconductors in 2010^[2,3]^ sparked a rapidly growing field for engineered photonic materials. In the monolayer limit, many TMDs are direct band gap semiconductors with an optical gap in the visible or near-infrared spectral range.^[4]^ The most outstanding features of 2D TMDs from this perspective are their sizeable band gaps, valley circular dichroism, pronounced excitonic effects with large binding energies, and controllable interlayer coupling. The material properties can be modified through doping, strain, external fields and by stacking arrangements of these van der Waals (vdW) materials, providing an attractive platform for applications in electronics, optoelectronics, and valleytronics,^[5,6]^ as well as for field-effect-transistor-based systems^[7]^ and quantum emitters.^[8]^

Both for fundamental reasons and for the implications for device design and performance, we seek a detailed understanding of the temporal evolution of exciton formation and dissociation, interfacial charge transfer and nonlinear polarization in symmetry-broken materials. This is particularly true for electronic and optoelectronic devices such as transistors, photodetectors, or solar cells, where charge carrier dynamics at the metal–semiconductor interface can determine the functionality of the device. Most commonly, time-resolved studies of exciton dynamics are performed using transient absorption measurements or second harmonic generation spectroscopy, requiring a significant degree of understanding of the spectroscopic signatures and associated relaxation channels to interpret the data. In contrast, time-domain THz emission spectroscopy (TES) represents a direct and nondestructive inspection probe of charge carrier dynamics in condensed-matter systems. It has been applied, to mention a few diverse examples, to study the dynamics of Dirac fermions,^[9]^ investigate plasmon-driven hot carriers,^[10]^ analyze transient photocurrents in layered materials,^[11–13]^ extract detailed information on charge transport and electron–phonon coupling in materials for photovoltaics,^[14]^ and evaluate light-induced currents at domain walls in ferroelectrics.^[15]^ THz emission upon photoexcitation by an ultrashort laser pulse is, in the majority of cases, explained by the creation of ultrafast photocurrents.^[16]^ The origin of the transient photocurrent can be manifold, including interfacial charge transfer, optical rectification, acceleration of charged particles due to a surface depletion field, or the diffusive carrier transport in the photo-Dember effect. Important information about these currents is encoded in the THz waveforms, including its polarization, amplitude, phase, and polarity, which in turn can reveal information such as the material’s structural symmetry, the direction of the current flow, charge transfer times, and carrier concentrations. Consequently, TES provides a very useful approach to examine charge carrier dynamics in emergent materials.

In this brief review, we first describe the experimental method of TES, in which we distinguish between different detection geometries, highlighting how one can discriminate between in-plane and out-of-plane photocurrents. We then discuss the present understanding of several distinct mechanisms that produce transient currents. In contrast to more general reviews on THz emission spectroscopy,^[17–21]^ here we focus on TES from vdW materials, as the manifold of energy band-dependent optical, magnetic, and electronic phenomena exhibited by these materials motivates the continued effort to understand charge dynamics and energy band alignment for charting new applications.

## Method

The first works on THz emission from semiconductor surfaces^[22,23]^ examined cases with built-in static electric fields occurring at surfaces or interfaces, providing a natural bias field for the motion of photogenerated carriers. After ultrafast laser excitation, photo-induced charge carriers are thus accelerated by the field to create a transient photocurrent. The pulse duration of the excitation pulse generally determines the rise time of the photocurrent, whereas the decay time corresponds to the carrier transit time across the field region (assuming the transit time remains shorter than the electron–hole recombination time).^[23]^ The relevant time scales are typically in the range from below a hundred femtoseconds to a few picoseconds, leading to radiated electromagnetic waves in the THz spectral range. More precisely, the THz electric field amplitude (in the far field) is proportional to the charge acceleration or time derivative of the photocurrent density

1 $$\begin{aligned} E_{\textrm{THz}} \propto \frac{\partial J}{\partial t}. \end{aligned}$$

**Figure 1:**
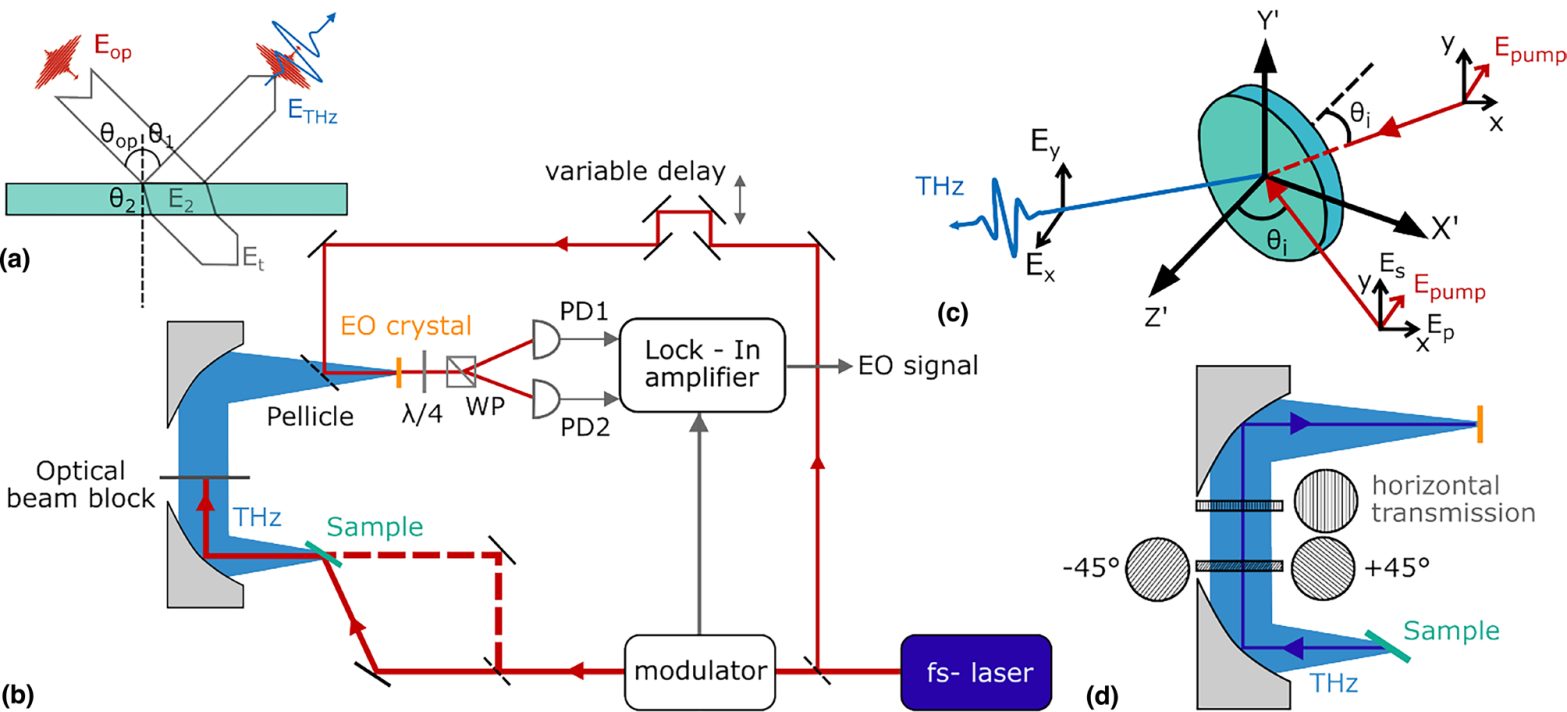
(a) Propagation direction of an incident optical beam, the outward-emitted THz field, and the inward-emitted field, all complying with the generalized Snell’s law. When electromagnetic beams are generated from a semiconductor surface, the outward radiated wave is collinear with the reflected optical beam in air, with $\theta _{\textrm{op}} \approx \theta _{1}$. (b) Sketch of a typical TES setup. (c) Schematic of electric field components when a semiconductor is excited in either reflection or transmission geometry. *x* and *y* denote coordinates in the lab frame, which aligns with the *p*- and *s*-polarized field components of the pump pulse, *X’, Y’*, and *Z’* refer to the crystal orientation axes, $\theta _i$ is the angle of incidence. (d) Diagram of wire-grid polarizers inserted into the beam path to extract the *p*- and *s*- polarized components ($\mathrm {E_x}$ and $\mathrm {E_y}$, respectively) of the THz waveform. *PD* photo diode, *WP* Wollaston prism, *EO* electro-optic.

We note that the emitted field can be additionally modulated by the frequency dependent dielectric constant,^[24]^ imprinting information about IR active phonons in the emitted THz spectrum.^[25]^ The electromagnetic wave from a thin layer of photocurrent near the surface emits in both transmitted and reflected directions, as sketched in Fig.1(a), with propagation directions following a generalized Snell’s law^[23]^ $n_1(\omega _{\textrm{op}} )\sin \theta _{\textrm{op}} \approx n_1 (\omega _{\textrm{THz}})\sin \theta _1 \approx n_2(\omega _{\textrm{THz}}) \sin \theta _2$, where $n_2(\omega _{\textrm{THz}})$ is the sample’s index of refraction for the radiated THz pulse, $n_1(\omega _{\textrm{op}})$ and $n_1(\omega _{\textrm{THz}})$ are the indices of refraction of optical pump and radiated THz pulse outside the sample. In air, we can assume $n_1(\omega _{\textrm{op}}) \approx n_1(\omega _{\textrm{THz}})$ and, hence, the outward radiated wave is collinear with the reflected optical beam, with propagation direction $\theta _{\textrm{op}} \approx \theta _{1}$. In addition to the motion of mobile carriers highlighted above and described as a current density from free charges ${\textbf{j}}_\mathrm{f}$, the total current density can include contributions from bound ${\textbf{j}}_\mathrm{b}$ current distributions^[20]^

2 $$\begin{aligned} {\textbf{J}} = {\textbf{j}}_{\mathrm{f}} + \textbf{j}_\mathrm{b} = \textbf{j}_\mathrm{f} + \frac{\partial {\textbf{P}}}{\partial t} + \nabla \times {\textbf{M}}. \end{aligned}$$

Here the bound current can be considered as arising from polarization ${\textbf{P}}$ and magnetization ${\textbf{M}}$ terms. Therefore, ${\textbf{j}}_\mathrm{b}$ provides sensitivity to emission associated with localized electrons, as well as phonons, time-dependent magnetization^[26–28]^ and ferroelectric polarization.^[29,30]^ The bold letters in Eq. (2) indicate their vector nature. In the following, we describe a typical TES experimental arrangement and further discuss different sources of transient photocurrents, their directionality, and the polarization properties of the radiated electromagnetic wave.

### Experimental arrangement

Figure 1(b) depicts a typical experimental arrangement for TES in reflection as well as in transmission mode. An ultrafast laser with femtosecond pulse duration is used as a pump source. It is divided by a beam splitter into a pump and a probe pulse for recording the THz waveform by electro-optic sampling (EOS), as described below. The pump pulse is chopped at a particular frequency, which is used as reference signal for lock-in detection of the THz waveform. The sample can be excited either in reflection [solid line in Fig. 1(b)] or transmission (dashed line) geometry, with the emitted THz pulse collected with a set of parabolic mirrors. A long-pass filter is used to block the optical pump pulse. The forward propagating THz waveform is detected by EOS using a nonlinear crystal (typically ZnTe or GaP), a quarter-wave plate, Wollaston prism, and balanced detection. In this scheme, the electric field of the THz pulse creates an anisotropy of the refractive index of the nonlinear crystal (by the Pockels effect) and thus alters the polarization state of the time-delayed probe pulse from linear to elliptical. The change in probe polarization creates an intensity asymmetry in the horizontal and vertical components of the probe pulse, which are split by the Wollaston prism and then detected with a balanced detector using a lock-in amplifier. The full THz waveform is then constructed by recording this signal as a function of the pump-probe delay time. Because ZnTe has a significantly larger EO coefficient than GaP (by approximately a factor of four), it is typically used to detect weak THz signals. However, the detectable bandwidth is limited by transverse-optical (TO) lattice vibrations of the EO crystal (around $5.3\ \textrm{THz}$ for ZnTe and $11\ \textrm{THz}$ for GaP), which lead to THz absorption and severe velocity mismatch between the THz and optical pulses near the resonance. Owing to its higher TO resonance frequency, GaP is better suited for measuring very short THz pulses.^[31]^

Figure 1(c) depicts both reflection and transmission configurations and corresponding field components, wherein the indices *s* and *p* refer, respectively, to the *s*- and *p*-polarized waveforms, aligned with *y*- and *x*- coordinates in the lab frame. If the radiated electromagnetic wave originates from an out-of-plane photocurrent, the emitted THz waveform -regardless of the excitation geometry- produces a *p*-polarized electric field, since the current has no projection along the *y*-direction. For a sample with isotropic absorption, the THz amplitude remains independent of the pump polarization once the Fresnel reflection losses have been taken into account. However, when the angle of incidence (in transmission geometry) is normal to the sample, electric-field components arising from an out-of-plane transient current cannot be observed, as the radiated field along the direction of an oscillating dipole is zero. In this configuration, only the THz electric field components generated by an in-plane photocurrent can be detected. The polarization of this THz electric field will generally reflect the crystal structure of the material being probed, as well as the pump polarization. For the case of TES in reflection geometry, an oblique angle of incidence is normally chosen and the emitted THz pulse can consist of both *s*- and *p*-polarized electric field components arising from in- and out-of-plane currents. The presence of a THz field component in *y*-direction indicates the existence of an in-plane photocurrent.

To determine the THz emission polarization, a pair of wire-grid polarizers can be placed in the THz beam path, as indicated in Fig. 1(d). A downstream polarizer, used as an analyzer, is set to transmit electric field components in the x-direction, aligning the transmitted field with the EO crystal axis and ensuring an independent measurement with respect to the EO crystal axis. In other words, because EOS is itself polarization sensitive, the downstream polarizer is needed to measure only the projection of the THz field component in the x-direction. Two sets of measurements are performed with the upstream polarizer oriented at either $+45^{\circ }$ or $-45^{\circ }$, with recorded THz waveforms denoted as $E_\mathrm {{+45^\circ }}(t)$ and $E_\mathrm {{-45^\circ }}(t)$. The *s*- and *p*-polarized THz field components can then be calculated as $E_\mathrm{s}(t) = E_\mathrm {{+45^\circ }}(t) - E_\mathrm {{-45^\circ }}(t)$ and $E_\mathrm{p}(t) = E_\mathrm {+45^\circ }(t) + E_\mathrm {-45^\circ }(t)$, enabling convenient polarization analysis of the THz radiation and determination of the direction of the transient photocurrent.

### Photocurrent mechanisms

In general, electromagnetic radiation can result from a combination of in- and out- of-plane transient currents, depending on the sample symmetry, angle of incidence, and pump polarization. In this perspective, we emphasize the association of different mechanisms of current generation with the direction of current flow, and how this, in turn, relates to the observed polarization dependence of the emitted THz radiation. We frame the discussion in terms of processes most relevant for measurements of TES in vdW materials.

**Figure 2:**
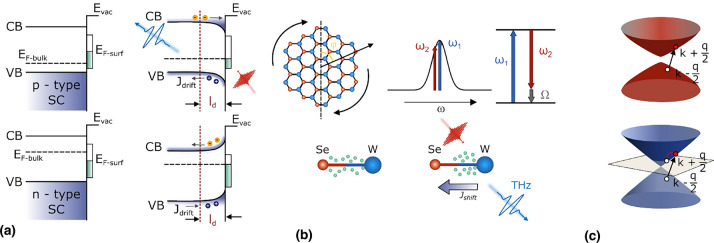
Representative mechanisms for THz emission. (a) Schematic diagrams of surface band bending, resulting in the acceleration of photoexcited carriers. The direction of the current flow depends on the doping type of the semiconductor (p- or n-type). (b) Representation of THz generation by (non-resonant) optical rectification (OR) (top) and by (resonant) shift current (bottom) as mediated by the second-order nonlinear response of the material. OR can be understood as coherent difference frequency generation of all spectral components within a broadband pump pulse. For excitation above the band gap energy, shift currents displace the charge density. (c) Schematic for the photon drag effect showing non-vertical photoexcitations in semiconductors (red) and semi-metals (blue) due to the presence of a finite photon-momentum **q**, adapted from Ref. [33]. The bright brown plane represents the Fermi surface. Abbreviations: *VB* - valence band; *CB* - conduction band; $E_\mathrm {F-bulk}$ - Fermi level of bulk material; $E_\mathrm {F-surf}$ - Fermi level of surface states; $E_{\mathrm{vac}}$ - Vacuum energy; $l_{\mathrm{d}}$ - depletion region; $J_{\mathrm{drift}}$ - drift current density.

A prominent mechanism for an out-of-plane transient photocurrent, described in early works on THz emission from semiconductor surfaces, is due to a surface depletion field, as sketched in Fig. 2(a). Surface states arising from the interruption of lattice periodicity can give rise to a narrow energy band within the bulk material’s band gap. In doped semiconductors, the Fermi level lies below the surface states for *p*-type doping and above them for *n*-type doping. Because of the energy difference between surface and bulk, either electrons or holes (depending on the doping type) will transfer from the bulk to the surface until the Fermi levels become aligned at equilibrium, resulting in band bending and a built-in electric field in this depletion region. When the semiconductor is excited with photon energies larger than the band gap, the built-in electric field accelerates photoexcited charge carriers, generating a time-dependent drift current that leads to THz radiation. At high pump fluences, the THz amplitude saturates due to a screening effect^[32]^. In this process, free electrons generated by the leading edge of the excitation pulse counteract the built-in field, reducing the flow of subsequently generated charge carriers. The emitted THz waveform is thus primarily shaped by the dynamical response of the photocurrent, the extent of the surface depletion field and the absorption characteristics of the optical pump and generated THz pulse. Consequently, because the generated photocurrent is highly sensitive to external factors such as adsorbed molecular layers or oxide growth at the surface, THz emission spectroscopy serves as a robust technique for probing local surface properties.

Note that, from our perspective, the concepts of a depletion field and “depletion width,” as defined for bulk semiconductors, are not suitable for describing THz emission processes in monolayer TMDs, since the electron wavefunction is localized within the transition metal layer, prohibiting out-of-plane electron displacement. In the limit of ultrathin vdW semiconductors, the concept of surface band bending remains complex and is strongly influenced by the substrate and dielectric environment, which can break the out-of-plane crystal symmetry. As a result, interface and electrostatic effects can significantly modify the drift contribution in 2D samples compared to their bulk counterparts.

While THz emission from above-gap excitation of wide gap semiconductors is regularly caused by Fermi-level pinning and an associated surface depletion field, narrow gap semiconductors often exhibit emission arising from the different diffusive motion of photogenerated carriers through the photo-Dember effect.^[16,19,34]^ A large disparity between electron ($\mu _{\textrm{e}}$) and hole ($\mu _{\textrm{h}}$) mobilities causes spatial separation of photoexcited carriers at the semiconductor surface, generating a net photocurrent through carrier diffusion. For $\mu _{\textrm{e}} > \mu _{\textrm{h}}$, electrons penetrate deeper into the crystal from the illuminated surface than holes, leading to the rapid formation of an electric dipole. The net transient Dember dipole lies perpendicular to the surface (for illumination of a broad area of a strongly absorbing semiconductor) and the THz radiation pattern is parallel to the surface.

In contrast to drift and diffusive photocurrents, which naturally occur predominantly in an out-of-plane direction and are insensitive to azimuthal rotations along an axis normal to the crystal surface, second-order nonlinear processes can cause in- and out-of-plane photocurrents, and can contribute to in-plane current densities in the case of low dimensional materials. The optical field of a pulsed laser source containing frequency components $\omega$ and $\omega + \Omega$ induces a nonlinear polarization in a non-centrosymmetric medium through the second-order nonlinearity as

3 $$\begin{aligned} P(\Omega ) \propto \chi ^{(2)}(\Omega )E(\omega )E^*(\omega +\Omega ), \end{aligned}$$

where $\Omega$ corresponds to the THz frequency, as indicated in Fig. 2(b). If the frequency dependence of $\chi ^{(2)}$ can be neglected, then this process gives rise to a polarization that varies in time with the laser intensity profile, hence the description of this process as optical rectification (OR). When charge carriers are excited above the optical band gap, shift and injection processes can lead to additional sources for transient currents. These processes are often termed collectively as *resonant* or *above-gap* rectification.^[35]^ The shift current is also known as the *bulk photovoltaic effect*.^[36]^ As the injection current is generated only by circular polarized light, it is also called the *circular photovoltaic effect*.^[37]^ In the following, we will only consider the shift current, as the injection current goes beyond the scope of this review article and, to the best of our knowledge, has not been investigated in TMDs. The second-order susceptibility tensor (ignoring explicit tensor notation) for $\Omega \ll \omega$ takes the form^[38,39]^

4 $$\begin{aligned} \chi ^{(2)}(\Omega ; \omega , -\omega +\Omega ) = \chi '^{(2)}(\Omega ; \omega , -\omega +\Omega ) + \frac{\sigma _2(\Omega ; \omega , -\omega +\Omega )}{-i\Omega }. \end{aligned}$$

The first term corresponds to the non-resonant OR process, while the second term is linked to the generation of carriers in resonantly excited bands, which gives rise to a current. The latter arises from shift currents that occur on femtosecond time scales during photo-excitation, wherein the center of charge is changing between initial and final states, producing a coherent shifting of the real-space charge density. The shift is on the order of a bond-length, as sketched in Fig. 2(b) for Se and W in $\textrm{WSe}_{2}$. Because the carrier current is related to the time derivative of the optically induced polarization, the rectification and shift currents take the form

5 $$\begin{aligned} & J_{\textrm{rect}}(t)= 2\epsilon _0 \chi '^{(2)} \frac{\partial }{\partial t } E(t)E^*(t)\end{aligned}$$

6 $$\begin{aligned} & J_{\textrm{shift}}(t) = 2\epsilon _0 \sigma _2 E(t)E^*(t), \end{aligned}$$

where the rectification current follows the time derivative of the optical pulse envelope and the shift current follows the envelope directly.^[39]^ Because shift currents originate from real electronic transitions, they can be observed only under above-band gap excitation. By contrast, rectification currents arise from the asymmetric oscillation of electrons within their bonds, involving virtual states, and can therefore occur for both above- and below-band gap excitation. For above-band gap excitation, however, shift currents typically dominate the DC response, often by orders of magnitude.^[40]^

In centrosymmetric crystals, second-order nonlinear optical effects can occur only through mechanisms that consider a finite momentum transferred from a photon to a free electron, potentially leading to the photon drag effect (PDE).^[41–43]^ Such a momentum transfer is allowed in all materials, independent of their symmetry, when the sample of interest is illuminated at oblique incidence. However, energy and momentum conservation dictate that for photons to transfer momentum to electrons (or holes), the lattice must be involved through electron–phonon interactions [see Fig. 2(c)]. The resulting momentum and, consequently, the drag current persist only for the duration of the momentum relaxation time, which is governed by carrier-phonon interactions. In other words, stronger electron–phonon coupling leads to a higher rate of momentum transfer but a shorter momentum relaxation time. Consequently, owing to their relatively low momentum relaxation rates (compared to semiconductor crystals), linear energy dispersion, and high carrier mobility, graphene-based materials are well-suited candidates for exhibiting the PDE.^[44]^ Notably, due to the centrosymmetric nature of graphene, second-order $\chi ^{(2)}$ nonlinearities, as described above, are forbidden, excluding mechanisms like OR or shift currents.

## TES on vdW materials

In the following, we review studies of TES in van der Waals materials. The discussion is divided into investigations of bulk (or near-bulk) materials and investigations of systems near the monolayer limit, including monolayers, few-layer materials, and heterostructures. For the bulk case, existing reports have focused on TMD crystals, while for atomically thin materials, both graphene and the TMDs have been examined.

### Bulk TMDs

The first works on THz surface emission from semiconducting TMDs were performed only a few years ago and examined $\mathrm {WS_2}$^[45]^ and $\mathrm {MoS_2}.$^[46,47]^ Despite their similar crystal structure and optoelectronic properties, they exhibit THz radiation with different dependences on the pump polarization angle, suggesting disparate mechanisms for the generation of the THz emission. All experiments were conducted at room temperature with a mode-locked Ti:sapphire regenerative amplifier (35 fs pulse duration and 1 kHz repetition rate). The linearly polarized femtosecond laser excitation with a photon energy of 1.55 eV is higher than the band gap energies of bulk $\mathrm {MoS_2}$ and $\mathrm {WS_2}$ (1.29 eV and 1.35 eV,^[45]^ respectively). TES was performed in transmission as well as reflection mode.

**Figure 3:**
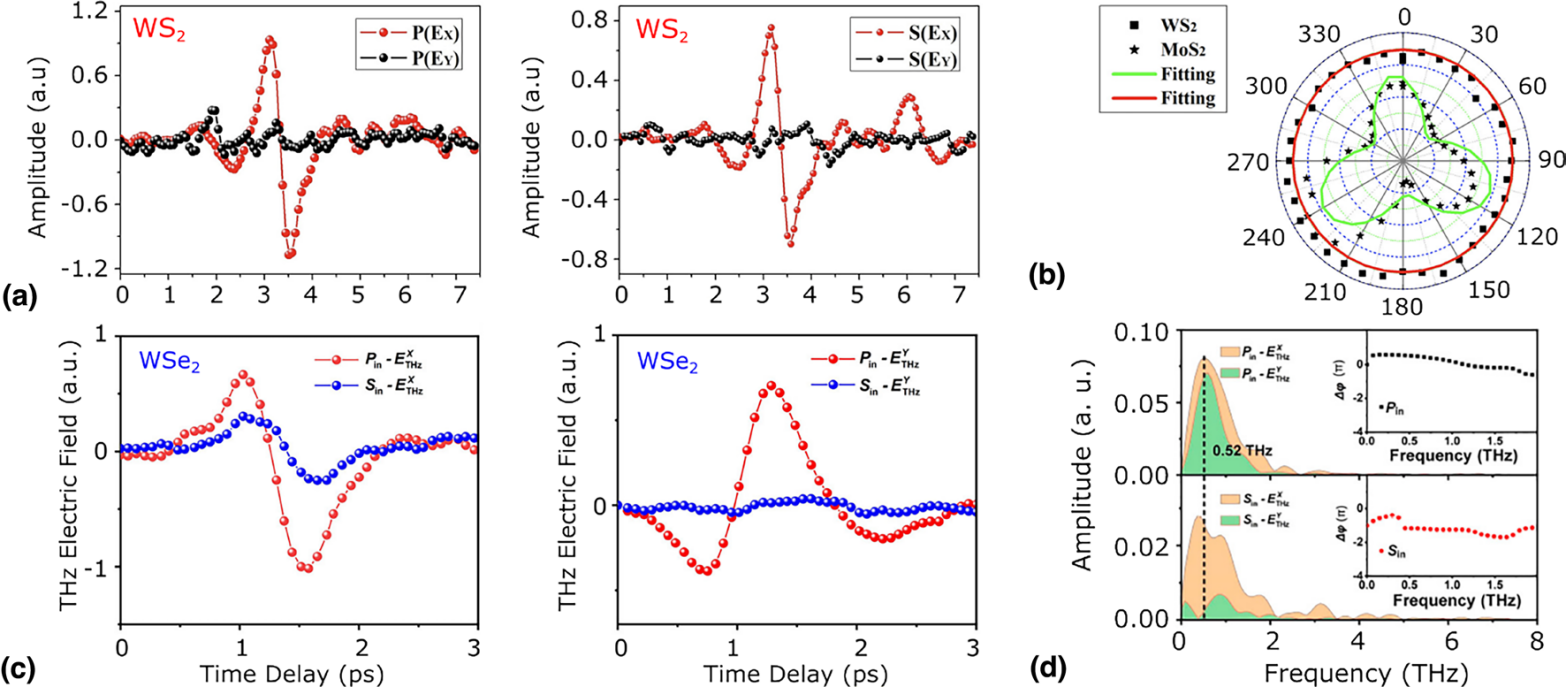
THz amplitudes with respect to the polarization of the excitation pulse and azimuthal angle. (a) THz field components $E_{\mathrm{x}}$ (red) and $E_{\mathrm{y}}$ (black) when bulk $\mathrm {WS_2}$ is excited with *p*- (left) and *s*-pol light (right). The absence of a THz field in the y- direction and insensitivity of waveform polarity to the pump polarization indicates an out-of-plane transient photocurrent. (b) THz amplitude with respect to the azimuthal angle, confirming a drift current for $\mathrm {WS_2}$ (red) and indicating in-plane surface optical rectification for $\mathrm {MoS_2}$ (green). (c) Emitted THz electric field $E_{\mathrm{x}}$ (left) and $E_{\mathrm{y}}$ (right) from a 7-layer thick $\mathrm {WSe_2}$ sample excited with *p*- (red) and *s*-pol (blue) light. The sensitivity of the THz waveform to the pump polarization state suggests contributions from an in-plane shift current and out-of-plane drift current to the emitted electromagnetic wave, leading to elliptically polarized THz fields, as shown in (d). (a) and (b) adapted and reprinted with permission from Ref. [45]. (c) and (d) adapted and reprinted with permission from Ref. [49].

Figure 3(a) shows the horizontal $E_x$ and vertical $E_y$ components of the outward radiating THz waveform when bulk $\mathrm {WS_2}$ is excited at a $45^{\circ }$ angle of incidence with a pump that is either *p*- or *s*-polarized. In both cases, the emitted THz field exhibits only components in the x-direction, indicating an out-of-plane current. The assumption is further confirmed by the insensitivity of the THz amplitude to the azimuthal angle [Fig. 3(b)] and vanishing THz radiation when the sample is pumped in transmission geometry at normal incidence. Due to similar electron and hole mobilities,^[48]^ the photo-Dember effect was discounted as a possible mechanism for the longitudinal photocurrent. Instead, THz radiation was attributed to photo-carriers accelerated in the built-in surface depletion field caused by Fermi-level pinning in the usually p- type semiconductor. The radiated THz amplitude demonstrates a saturation effect with the pump fluence, which is attributed to screening of the surface depletion field. Similar results of a drift current caused by a surface depletion field were reported for bulk $\mathrm {WSe_2}.$^[49]^

In contrast, $\mathrm {MoS_2}$ demonstrates a strong $3\varphi$ dependence on the azimuthal angle [Fig. 3(b)] and exhibits an electric field component in y-direction, suggesting an in-plane photocurrent from a second-order nonlinear process. This is further confirmed by TES measurements in transmission mode. While the THz peak amplitude decreases as the incidence angle changes from $-40^{\circ }$ to $0^{\circ }$, consistent with THz emission originating from an out-of-plane photocurrent, a clear THz signal is still observed from $\mathrm {MoS_2}$ under normal-incidence excitation, indicating that resonant optical rectification may contribute to THz emission. Although bulk $\mathrm {MoS_2}$ is centrosymmetric, optical rectification can still take place because the symmetry is broken at the surface, exhibiting threefold rotational symmetry.^[46]^ The difference in azimuthal angle dependence between $\mathrm {WS_2}$ and $\mathrm {MoS_2}$ is revealed by first-principle calculations of the nonlinear optical coefficient. The $3\varphi$ dependence on the azimuthal angle is dominated by the $d_{22}$ tensor element of the nonlinear optical coefficient, which is predicted to be more than seven times larger for $\mathrm {MoS_2}$ (3.8 pm/V) than for $\mathrm {WS_2}$ (0.5 pm/V) at 1.55 eV.^[45]^ The significant difference is mainly attributed to the different density of states in the two materials at the photon excitation energy.

In 2020, coherent elliptically polarized THz radiation was reported on a $\sim$ 7 layer thick, polycrystalline $\mathrm {WSe_2}$,^[50]^ with an approximate indirect band gap of 1.3 eV. Because the THz waveform is sensitive to the polarization of the 1.55 eV excitation pulse [see Fig. 3(c)], the authors claim a contribution to the THz emission from a second-order nonlinear process, attributed to a shift current. Since the horizontal field component $E^{\mathrm{X}}_{\mathrm{THz}}$ of the radiated THz pulse is caused by a combination of in-plane shift current and out-of-plane drift current (due to the surface depletion field), it is naturally larger than the vertical field $E^{\mathrm{Y}}_{\mathrm{THz}}$, which can only originate from an in-plane shift current at oblique incidence. As the output THz emission can be reconstructed as $E(t) = E^\mathrm{X}_\mathrm{THz} \hat{e}_x + E^\mathrm{Y}_\mathrm{THz} \hat{e}_y$, induced by a shift and drift photocurrent, respectively, the electric field components exhibit a phase shift $\Delta \phi$ resulting in elliptical THz radiation [see Fig. 3(d)]. The ellipticity of the THz waveform can thus be readily controlled with a half wave plate for the pump pulse.

Nevinskas et al.^[51]^ further investigate THz emission from photoexcited bulk $\mathrm {MoS_2}$, $\mathrm {MoSe_2}$, and $\mathrm {WSe_2}$ (with a thickness of a few tens of $\mathrm {\mu m}$) with respect to the excitation wavelength of the femtosecond optical pulse. The samples are irradiated with a tunable photon energy from 1.3 to 2.4 eV and pump fluence on the order of $\sim 1\ \mathrm {\mu J/cm^2}$. They observe a gradually increasing THz emission starting at photon energies equal to the indirect gap and maximum radiation at photon energies corresponding to the direct band gap observed in bulk materials. The authors hypothesize that below the direct energy gap, carriers are excited from defect states, resulting in unbound electron–hole pairs, which further induce an ultrafast photocurrent surge in the surface field. In turn, for excitation above the direct band gap, it is speculated that excitonic effects can play a dominant role in THz emission.

**Figure 4:**
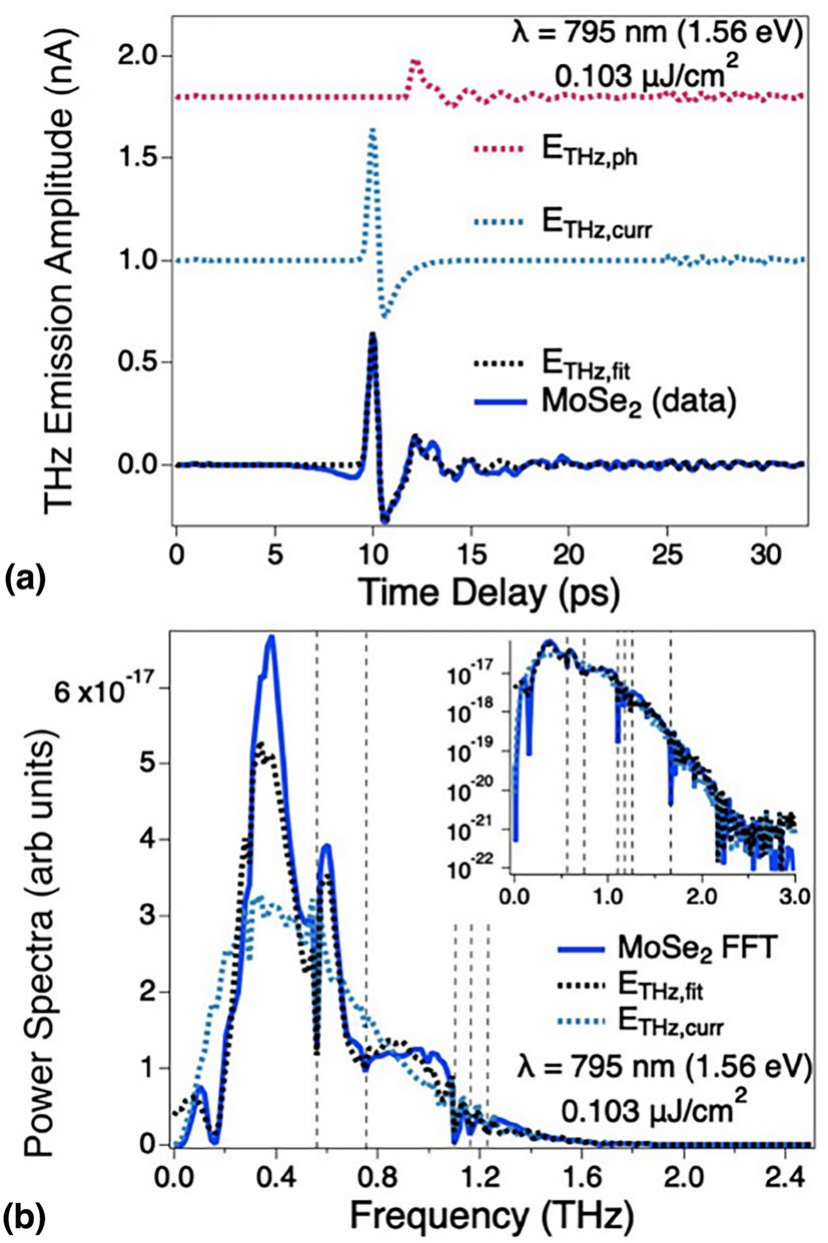
(a) THz emission from $\mathrm {MoSe_2}$, decomposed into $E_{\mathrm{THz,curr}}$ corresponding to a photo-induced transient current and $E_{\mathrm{THz,ph}}$ related to interlayer phonons. (b) Corresponding Fourier transformation of (a). Reprinted with permission from Ref. [52].

THz emission spectroscopy was further utilized to investigate coherent phonons in layered $\mathrm {MoSe_2}$ and $\mathrm {WSe_2}$.^[52]^ Afalla et al. employed pristine single crystal 2*H*-$\mathrm {MoSe_2}$ and 2*H*-$\mathrm {WSe_2}$ with thicknesses in the range of tens of micrometers, grown by the physical vapor transport method.^[53]^ Figure 4 depicts the time-domain and Fourier transformed THz emission when $\mathrm {MoSe_2}$ is excited in reflection geometry with a $\sim 100\ \textrm{fs}$ pulse, centered at $795\ \textrm{nm}$ ($1.56\ \textrm{eV}$), under open-air laboratory conditions. The excitation pulse is strongly absorbed due to the A-gap at the K point, resulting in efficient carrier excitation and high carrier mobility. The time-domain THz waveform can be decomposed into two contributions: (i) a current-driven THz emission component $E_\mathrm{THz,curr}$ which exhibits a peak THz amplitude at a time delay of approximately $10\ \textrm{ps}$ and is attributed to a photoinduced transient current generated by a surface depletion field; and (ii) subsequent oscillatory features which are attributed to interlayer phonon emission $E_\mathrm{THz,ph}$, and residual effects of water vapor. Since coherent lattice vibrations that emit THz radiation are IR-active,^[54,55]^ it is suggested that the low frequency components of the oscillatory features originate from IR coherent interlayer phonon modes. The phonon response emerges at the trailing edge of the current-driven THz emission at a time delay of $t_{\textrm{ph}} \sim 11.18\textrm{ps}$. In the frequency domain [see Fig.4(b)], the peaks and dips indicate constructive and destructive interference between $E_\mathrm{THz,curr}$ and $E_\mathrm{THz,ph}$. Analysis of the time-domain THz waveforms yields phonon frequencies of $f_1 = 0.357\ \textrm{THz}$ and $f_2 = 0.599\ \textrm{THz}$, where the lower frequency $f_1$ is tentatively assigned to a layer-breathing mode, and $f_2$ to an interlayer shear mode.

### Low dimensional vdW materials

**Figure 5:**
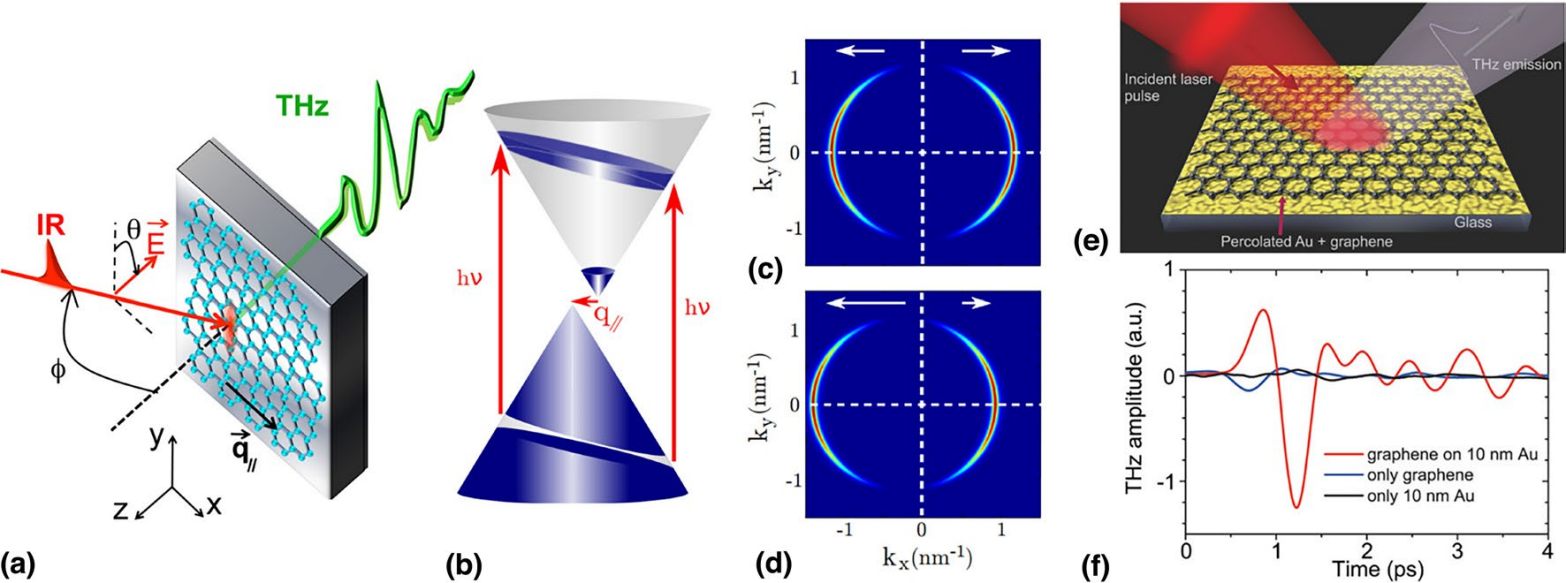
(a) Schematic of femtosecond pump pulse illumination of multilayer graphene, causing an in-plane transient photon drag current, emitting THz radiation. (b) Sketch of the anisotropic electron population distribution in momentum space after laser excitation at oblique incidence, $\mathrm {q_{\parallel }}$ depicts a finite momentum transferred from a photon. (c) Nonequilibrium electron distribution after excitation at normal incidence and (d) at oblique incidence. (e) Excitation of graphene deposited on a percolating gold layer. (f) THz waveform, emitted from a single layer graphene on a percolating Au film (red), only graphene (blue), and only percolated Au film (black). (a)–(c) reprinted with permission from Ref. [56]. (e) and (f) reprinted with permission from Ref. [57].

THz emission from unbiased graphene was first reported in 2014^[44]^ from single- and multi-layered graphene samples (1 $\sim$ 40 layers), deposited on dielectric substrates (either $\mathrm {SiO_2}$ or polymer). Within the experimental limitations, no qualitative dependence on either the substrate or sample thickness was observed. This was interpreted as an indication that carrier dynamics on the sub-picosecond time scale are primarily governed by intralayer processes, whereas at longer times, they are dominated by interlayer coupling. Studies on pump polarization and angle of incidence with linearly polarized visible or near IR excitation reveal an in-plane photocurrent, attributed to the photon drag effect (PDE). Maysonnave et al.^[56]^ further examine the PDE in multilayer (35-40 layers) graphene to elucidate a microscopic model. Under normal excitation, the nonthermal electron and hole distributions remain symmetric about the Dirac cone center [see Fig. 5(c)]. However, as indicated in Fig. 5(b) and (d), the population distribution at oblique incidence exhibits a shift relative to the center of the Dirac cone, enabling a net transient current. By including the next-nearest-neighbor coupling into their model, they conclude that THz emission arises from the unequal acceleration and damping of photogenerated electron and hole distributions. Bahk et al.^[57]^ further demonstrate that surface plasmons, excited by femtosecond laser pulses on either continuous or discontinuous gold substrates, significantly enhance THz emission from single layer graphene. In the case of graphene on a continuous $\sim 44\ \textrm{nm}$ gold substrate, the sample is excited with a $\sim$ $45^{\circ }$ angle of incidence for which the evanescent wave field couples resonantly with the propagating surface plasmon modes. At this so called surface plasmon resonance, the emitted THz field amplitude is enhanced by $\sim$ 30 times (compared to THz emission from graphene on glass), facilitated by the strongly localized electric field of surface plasmons excited near the gold surface, intensifying interaction between the pump pulse and graphene. In turn, in the case of graphene on a $\sim 10\ \textrm{nm}$ percolating gold film [see Fig. 5(e) and (f)], the authors attribute the enhanced emission of the x-component of the THz field to a longitudinal symmetry breaking induced by the presence of the Au film. The introduced second-order nonlinearity enables the excitation of localized surface plasmon “hot spots,” thereby enhancing optical rectification of the optical pulses.

The first works on THz emission from monolayer TMDs were conducted using a polycrystalline $\mathrm {WS_2}$ sample,^[58]^ grown on a sapphire substrate by chemical vapor deposition.^[59]^

This direct band gap material ($\mathrm {E_g} = 2.1\ \textrm{eV}$^[60]^) is excited in transmission mode at a $45^{\circ }$ angle of incidence with a femtosecond Ti:Sapphire system of varying polarization. Because the pump photon energy (1.55 eV) lies well below the optical gap, the THz emission mechanism was assumed to be dominated by (non-resonant) optical rectification. The THz amplitude exhibits a $2\alpha$ dependence on the pump polarization angle for linear polarization, but is insensitive to the azimuthal angle of the sample. The authors attribute the insensitivity to the azimuthal angle to the uniformly random distribution of $\mathrm {WS_2}$ triangular islands. Counterintuitively, they claim that the emitted electric field can be therefore expressed as $E_{\textrm{THz}} \propto Kd_{22}$, where *K* is a constant related to the pump intensity and $d_{22}$ is the nonlinear optical coefficient.

Terahertz surface emission from $\mathrm {MoSe_2}$ at the monolayer limit was first explored by Fan et al. in 2020^[61]^ and further studied for bilayer and multilayer $\mathrm {MoSe_2}$ by Yagodkin et al.^[62]^ Since the band gap of $\mathrm {MoSe_2}$ varies from 1.1 eV to 1.5 eV from bulk to monolayer (as a function of the number of layers),^[63]^ a Ti:Sapphire system with a photon energy of 1.55 eV is sufficient to drive above gap excitations in both material systems. Because charge carrier mobilities and the nonlinear optical coefficient differ between bulk and monolayer $\mathrm {MoSe_2}$, Fan and co-workers probed THz surface emission in both crystal configurations for comparison. To elucidate the contribution from nonlinear polarization to the emitted radiation, they study the effect of the crystalline orientation on the emitted THz waveform, revealing an insensitivity to the azimuthal angle for both bulk and monolayer $\mathrm {MoSe_2}$. For the monolayer sample, the observation is argued by citing Ref. [58] suggesting an "azimuthal independent" THz emission governed by $E_{\textrm{THz}} \propto Kd_{22}$. Overall, they ascribe the observed THz emission to a strong photocurrent induced by a surface depletion field and a weak current caused by surface optical rectification.

**Figure 6:**
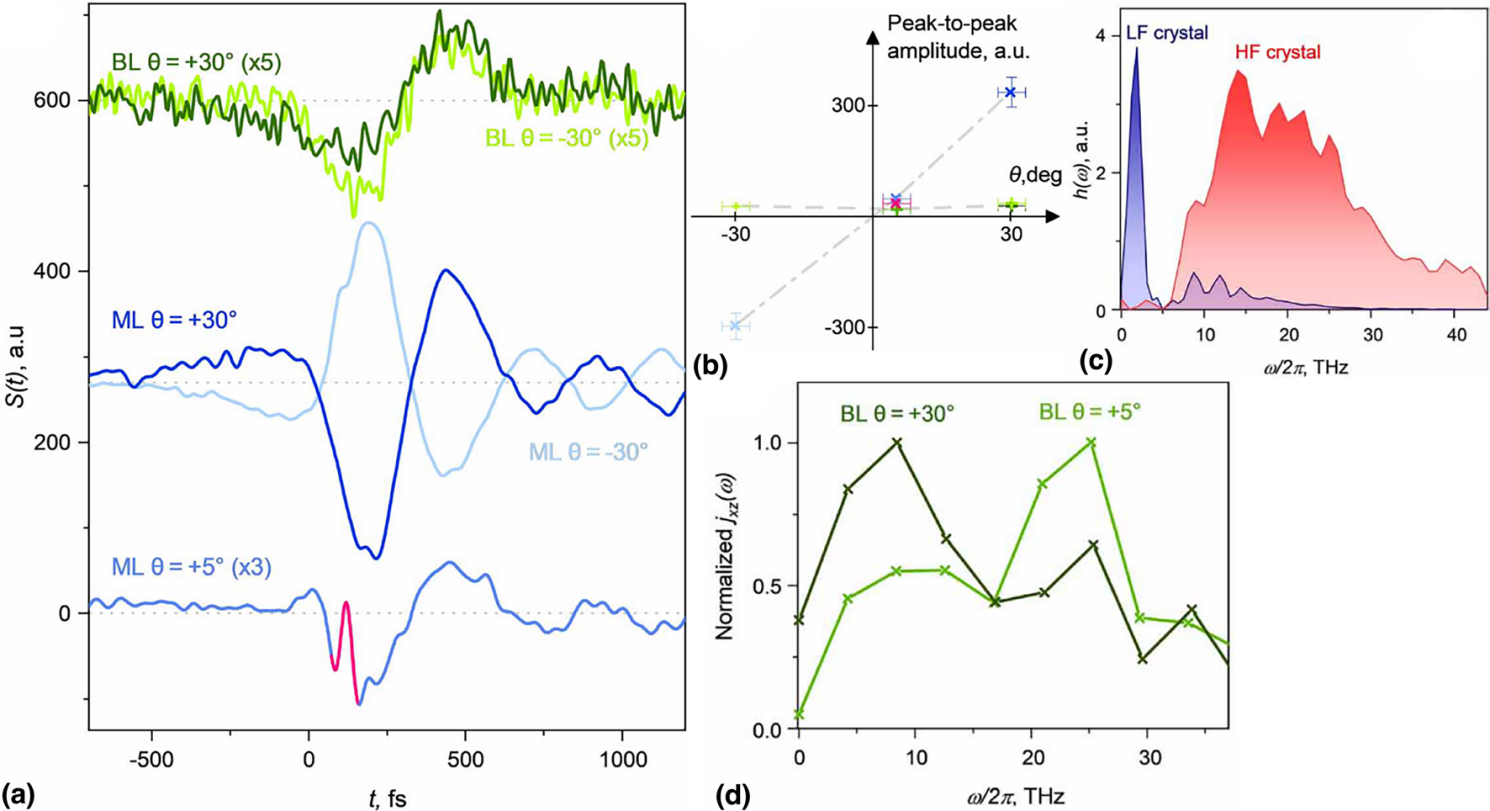
(a) THz waveform emitted by bilayer (green) and multilayer (blue) $\mathrm {MoSe_2}$ for different sample tilt angles (i.e., angle of incidence) measured with the low frequency (LF) detection scheme [see also (c)] and (b) corresponding peak-to-peak amplitude as a function of the tilt angle $\theta$. (c) Transfer function $h(\omega )$ of EOS for low- and high-frequency (LF and HF) detection crystals. (d) Fourier amplitude spectrum of the charge current in the bilayer sample for $+30^{\circ }$ and $+5^{\circ }$ tilt angle, extracted from the THz waveform measured with the HF detector. Reprinted with permission from Ref. [62].

In contrast, Yagodkin et al.^[62]^ detect components of in-plane photocurrents in a bilayer (BL) as well as in a multilayer (ML) $\mathrm {MoSe_2}$ sample and elucidate their origin. Their ultrafast laser (30 fs pulse width, 1.51−1.7 eV spectral bandwidth, centered at 780 nm, 80MHz repetition rate) in combination with a broadband high frequency detection scheme [see Fig. 6(c)] allows them to observe phenomena with much higher time resolution.

They excite their samples with a photon energy above the band gap of $\mathrm {MoSe_2}$ in a transmission geometry and record the THz waveform with EOS utilizing either a 1 mm or $10\ \mathrm {\mu m}$ thick ZnTe crystal, covering a spectral range of 0.8−2.5 THz and 10-30 THz, respectively. Figure 6(a) displays THz waveforms of BL and ML $\mathrm {MoSe_2}$ for different tilt angles of the sample, which is equivalent to changing the angle of incidence. The THz electric field is sampled with a 1 mm ZnTe crystal, detecting only low frequency components. The BL sample exhibits in-plane transient currents as demonstrated by the comparable THz waveforms for tilt angles of $\theta = +30^{\circ }$, $-30^{\circ }$ and $+5^{\circ }$. In turn, the polarity of the THz waveform from the ML is reversed, while maintaining its amplitude for $\theta =+30^{\circ }$, $-30^{\circ }$, indicating an out-of-plane photocurrent caused by a surface depletion field. However, the non-vanishing component at $\theta = +5^{\circ }$ suggests a quickly responding contribution [highlighted in pink in Fig. 6(a)] of in-plane origin. To understand the source of these fast dynamics, the THz electric field is sampled with the broadband $10\ \mathrm {\mu m}$ thick ZnTe crystal. The THz emission from a BL sample is assigned to a low frequency (centered around 8 THz) resonant shift current and noticeable oscillations at about 23 THz, as shown in Fig. 6(d). The authors suggest that the high frequency and fast decay constant of 55 fs of the photocurrent oscillations are related to quantum beats between two states separated by the corresponding energy ($23\ \textrm{THz} \overset{\wedge }{=}95\ \textrm{meV}$) and coherently excited by the pump.

**Figure 7:**
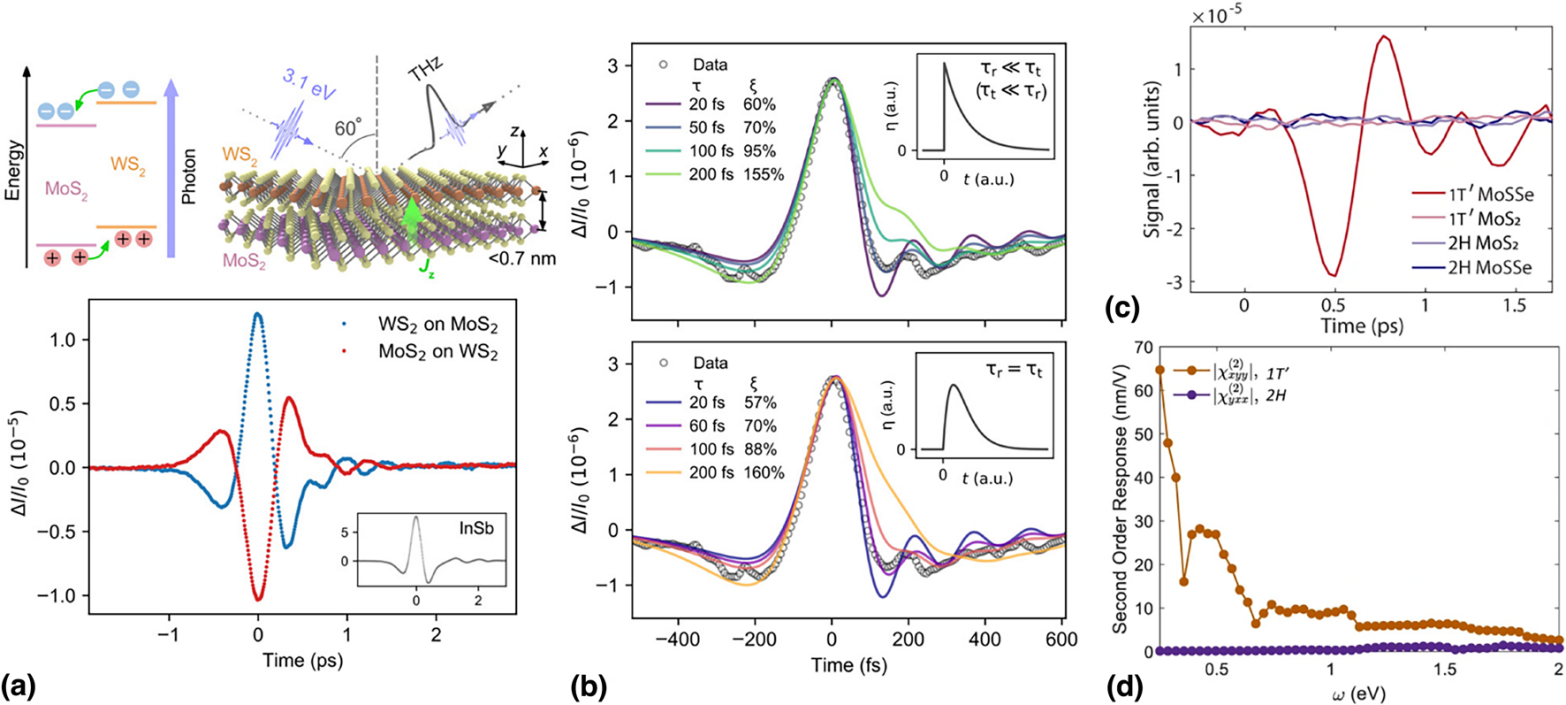
(a) Schematic energy bands in a $\mathrm {WS_2/MoS_2}$ heterostructure forming type-II band alignment, and interfacial current $J_z$ emitting a THz pulse after ultrafast laser excitation. The THz waveforms from $\mathrm {WS_2}/\mathrm {MoS_2}$ (blue) and $\mathrm {MoS_2}/\mathrm {WS_2}$ (red) confirm hole transfer from $\mathrm {MoS_2}$ to $\mathrm {WS_2}$, evident by the polarity of the THz waveform from a bulk InSb crystal as shown in the inset. (b) Measured THz waveform overlaid with simulations revealing relaxation time $\tau _r$, transfer time $\tau _t$, material response $\tau = \tau _r + \tau _t$ and transfer efficiency $\xi$. The inset shows the corresponding impulse response of interfacial current $\eta$. (c) THz waveforms from several monolayer TMDs, demonstrating an extensive enhancement in THz emission from Janus 1*T’*MoSSe samples. (d) Theoretically predicted second-order optical response of 1*T’*MoSSe (brown) and 1*H*MoSSe (purple) with respect to the excitation frequency $\omega$. (a) and (b) reprinted with permission from Ref. [64]. (c) and (d) reprinted with permission from Ref. [65].

A different type of 2D sample is that formed by a heterostructure of 2D semiconductors. For the case of heterostructures with type-II band alignment, it is known that rapid charge transfer will occur. This can be understood as the creation of a rapid transient current between the monolayers. In some sense this can be regarded as a digitized version of carrier flow at the surface of a bulk material driven by band bending, as discussed above. THz emission from this type of sample was demonstrated by Ma et al.^[64]^ They examined heterostructures of monolayer $\mathrm {MoS_2}$ and $\mathrm {WS_2}$, which are known to exhibit type-II band alignment when placed in contact with one another. A staggered energy band structure is thereby formed by the valence band maximum in $\mathrm {WS_2}$ and conduction band minimum in $\mathrm {MoS_2}$, enabling separation of photogenerated electron and holes into different layers which generates a net transient current across the interface [see Fig. 7(a)]. The optical pump is provided by the second harmonic of a Ti:Sapphire system, with a photon energy of 3.1 eV well above the band gap of both materials, initially creating hot carriers. Heterostructures with both stacking orders were fabricated on top of a sapphire substrate from large-area polycrystalline monolayers grown by chemical vapor deposition. Significant THz emission from the isolated monolayers of either material was ruled out prior by separate measurements on these samples. Figure 7 shows the emitted THz waveforms from $\mathrm {MoS_2}$/$\mathrm {WS_2}$ and $\mathrm {WS_2}$/$\mathrm {MoS_2}$ stacks with reversed polarity for the flipped sample geometry. The inset displays THz emission from a reference InSb sample, which is known to emit THz radiation due to the photo-Dember effect with electrons diffusing deeper into the bulk material. Comparison of these signals confirms that in the heterostructure electron transfer from $\mathrm {WS_2}$ to $\mathrm {MoS_2}$ (or hole transfer from $\mathrm {MoS_2}$ to $\mathrm {WS_2}$) is observed. The charge carrier dynamics involving both intralayer cooling at a rate $1/\tau _{\textrm{r}}$ and interlayer charge transfer at a rate $1/\tau _{\textrm{t}}$ were inferred from the temporal evolution of the THz pulse. For the applied model, the net interfacial current is further scaled with an efficiency factor $\xi$ accounting for loss channels, such as radiative recombination. The best fit in Fig. 7(b), including consideration of the amplitude of the THz waveform, suggests an interfacial transfer time in the order of 50 fs. As mentioned earlier, the pulse duration of the excitation pulse generally determines the rise time of the generated photocurrent, whereas the decay time corresponds to the carrier transit time. With a pump pulse duration of $\sim 40\ \textrm{fs}$, the estimated transfer time is close to the limit of the instrument response function. Such a rate of interfacial charge transfer is compatible with complementary optical pump probe measurements in Ref. [66].

Interfacial dynamics recorded by THz emission can be further exploited for sensing applications or to investigate molecular adsorption and desorption dynamics at 2D/3D vdW interfaces.^[67]^ For instance, Sano et al.^[68]^ observed that the shape and polarity of a THz waveform emitted from graphene-coated InP depends on the ambient gas, laser exposure time, and UV illumination - an effect attributed to band structure changes in the InP surface depletion layer caused by dipoles from adsorbed oxygen. Bagsican et al.^[69]^ extended this approach to graphene-coated surfaces on several semiconductors (InP, GaAs, and InAs) to study the effect of $\mathrm {O_2}$ adsorption on the charge carrier dynamics at the interface. THz emission was found to be differently affected by $\mathrm {O_2}$ adsorption due to distinct THz generation mechanisms in these semiconductors. Similar observations were made for $\mathrm {WS_2}$ spin-coated on InP,^[70,71]^ where temperature dependent measurements on THz emission allowed the authors to estimate the adsorption energy of oxygen molecules on 2D $\mathrm {WS_2}$ ( $\sim$ 0.24 eV). These findings suggest that THz emission from 2D/3D vdW materials can be used for probing surface chemical reactions (e.g., oxidation).^[69]^

Recently, Shi et al.^[65]^ reported on giant room-temperature nonlinearities in monolayer Janus TMDs in the 1*T’* phase, fabricated via an atomic-layer substitution method^[72]^ from 1*T’* $\mathrm {MoS_2}.$^[73]^ In their studies, they compare pristine $\mathrm {MoS_2}$ with Janus MoSSe in their 2*H* phase with a trigonal prismatic structure and distorted octahedral 1*T’* phase, respectively. The latter exhibit nontrivial topological properties featuring an inverted band gap in the THz range (on the order of tens of meV).^[74]^ Figure 7(c) shows emitted THz waveforms when the monolayer samples are excited in reflection geometry with 800 nm pulses. As 1*T’* $\mathrm {MoS_2}$ has a centrosymmetric structure, which excludes OR as a possible THz generation mechanism, no detectable THz signal is observed. The authors further assign the small THz amplitude from 2*H* $\mathrm {MoS_2}$ to a weak surface photocurrent. Polarization analysis of the emitted THz waveform from 1*T’* MoSSe reveals a strong *p*-polarized electric field component, accompanied by a weaker *s*-polarized component, indicating both in- and out-of-plane photoresponses. Theoretical results for the second-order susceptibility $\chi ^{(2)}$ are shown in Fig.7(d) for 1*T’* and 1*H’* MoSSe. The authors link the enormous nonlinearities at THz frequencies of 1*T’* MoSSe to the broken inversion symmetry caused by S and $\mathrm {S_e}$ atoms, and topological band mixing, which induces wavefunction hybridization and thereby enhances the overlap of electron and hole wavefunctions near the band edges of valence- and conduction band. These results suggest that such material systems engineered to strong inversion symmetry breaking may be a promising route to reach THz/infrared sensing on the nanoscale using atomically thin materials.

## Summary and outlook

Ultrafast carrier dynamics in prototypical semiconductor TMDs, such as $\mathrm {MoSe_2}$, $\mathrm {MoS_2}$, $\mathrm {WSe_2}$, and $\mathrm {WS_2}$, have been the subject of many recent studies,^[75–79]^ typically employing time-resolved absorption/reflection or optical emission spectroscopy to probe the time evolution of the system. As the studies reviewed above have illustrated, TES spectroscopy can provide complementary information by revealing the direction, magnitude, and dynamics of the currents produced by spatial motion of the carriers. As a result, TES has become a valuable tool for probing ultrafast current dynamics in emerging materials. Foundational work on THz emission spectroscopy has already been performed to understand and identify microscopic mechanisms leading to the observed photocurrents and their dynamics in bulk TMDs. In $\mathrm {WS_2}$^[45]^ and $\mathrm {WSe_2},$^[49]^ THz emission has been attributed to out-of-plane drift currents caused by a depletion field, while in $\mathrm {MoS_2}$^[46,47]^ and $\mathrm {MoSe_2}$^[61]^ transient photocurrents have been described as arising from a combination of drift currents and in-plane optical rectification. For $\mathrm {p-MoS_2}$, $\mathrm {n-MoSe_2}$, and $\mathrm {p-WSe_2}$ the situation seems to differ when the intrinsically doped semiconductors are pumped with excitation energies above or below the direct band gap of the bulk material.^[51]^ Recently, THz emission from coherent phonon oscillations has been reported in layered $\mathrm {MoSe_2}$ and $\mathrm {WSe_2}.$^[52]^ Thus, while considerable progress has been made on identifying different mechanisms for THz emission that may be operative in different materials, an overall picture of their relative importance in different materials under different excitation conditions is still to be obtained. The physics of THz generation can change significantly in the 2D limit of vdW materials, where TMDs become direct band gap semiconductors, and exhibit a non-centrosymmetric crystal structure for an odd number of layers. To date, only a few publications examine charge carrier dynamics in low dimensional vdW materials with TES. In single and multilayer graphene, THz emission is found to arise from the photon drag effect,^[44,56]^ which can be further enhanced by surface plasmons on a gold substrate.^[57]^ A resonant shift current is observed in bilayer $\mathrm {MoSe_2}$^[62]^ with fast oscillating features attributed to quantum beats between coherently excited inter- and intralayer excitons. TES on bilayer TMD heterostructures have been conducted by Ma and co-workers,^[64]^ demonstrating an ultrafast current flow across a vdW interface. Studies on charge carrier dynamics on 2D vdW/3D semiconductor interfaces reveal potential application to probe surface chemical reactions with THz emission spectroscopy.^[68–71]^ Recently, TES has been applied for the first time on a monolayer Janus topological semiconductor,^[65]^ revealing giant room-temperature nonlinearities, especially in the THz frequency domain.

The summarized works in this prospective article demonstrate the great potential of TES to investigate linear and nonlinear optical response, charge carrier dynamics, coherent phonon oscillations, energy band structures, and exciton-exciton interactions in TMDs, in particular in the low dimensional regime. However, this exciting new research area seems to be still in an early stage, as the amount of published work is readily comprehensible, which will undoubtedly change within the next few years. For example, after the pioneering work on interfacial charge transfer in 2D TMD heterostructures, a possible next step would be to examine the sensitivity of charge transfer dynamics on the twist angle or interlayer sliding between two monolayers. Moreover, opto-electronic devices, such a transistors or photodetectors based on low dimensional TMDs came to prominence within last few years. Understanding energy band bending and its correlated charge carrier dynamics at metal–semiconductor interfaces is at the heart of opto-electronic device design, and would be conveniently accessible with TES. Although studies on molecular adsorption and desorption dynamics at 2D/3D vdW interfaces have already been performed ten years ago, the effect on substrate dependent THz emission and correlated photon-induced oxidation as well as degradation of the 2D vdW material is yet missing. First results on 1*T’* MoSSe pave the way for Janus-TMD based THz detectors, yet rigorous studies on the excitation wavelength and further Janus materials are missing, albeit a theoretically predicted giant second-order optical response in the mid-IR spectral range. To the best of our knowledge, THz emission in 3*R* stacked TMDs has not yet been demonstrated, despite the intrinsic out-of-plane polarization and consequent bulk photovoltaic effect. TES would allow to determine polarization strength and direction of these materials. In general, recent works on TES in TMDs have only unleashed the great potential in combining these promising fields, that is the controllability of optical and electronic properties in low dimensional vdW materials and a direct probe to measure their charge carrier dynamics and optical response with TES.
